# COVID-19-associated familial acute disseminated encephalomyelitis (ADEM): A case report

**DOI:** 10.1016/j.idcr.2021.e01264

**Published:** 2021-09-01

**Authors:** Sivaram Neppala, Dinesh Kumar Sundarakumar, Joseph W. Caravella, Himaja Dutt Chigurupati, Prateek Patibandla

**Affiliations:** a Department of Inte\rnal Medicine, University of Incarnate Word at Laredo Medical Center, Laredo, TX, 78041 USA; b Department of Interventional Radiology, University of Incarnate Word at Laredo Medical Center, Laredo, TX, 78041 USA; c Department of Internal Medicine, Reading Hospital, West Reading, PA 19611, USA

## Abstract

**Background:**

Several neurological complications are being reported in hospitalized patients with severe COVID-19 infection. This is presumed due to direct spread of infection or due to immunological response. Acute disseminated encephalomyelitis (ADEM) is a rare inflammatory and demyelinating disorder of the central nervous system that is often preceded by infection or vaccination. Very few cases of ADEM have been reported in the literature that are associated with COVID-19 infection.

**Case Report:**

Here we demonstrate familial cases of ADEM in a hospitalized father and son, who presented to the emergency department with fever and shortness of breath, later diagnosed with COVID-19, and subsequently requiring mechanical ventilation. Both patients developed neurological symptoms with upper motor neuron involvement at approximately day 30 of admission. MRI of the brain demonstrated bilateral multifocal periventricular white matter FLAIR signal hyperintensities consistent with ADEM. The patients were treated with medium dose IV methylprednisolone with variable outcomes. The 49-year-old son developed severe residual neurological deficits with encephalomalacic changes on MRI which required extensive rehabilitation; meanwhile, the 68-year-old father predominantly had pulmonary sequelae including fibrosis and the development of a pneumatocele, but he had a better neurological outcome.

**Conclusion:**

To our knowledge, this is the first reported case report of ADEM involving father and son in severe COVID-19 infection. Final neurological outcomes in these patients appeared to be in line with the severity of COVID-19 infection. More research is needed to better understand the management of ADEM in patients with severe COVID-19 infection.

## Introduction

Acute disseminated encephalomyelitis (ADEM) is a rare inflammatory demyelinating disorder that usually manifests one to three weeks following a viral infection or vaccination [11], [12]. ADEM is most common in children and young adults [16], [17] but very few cases have been reported in middle-aged and elderly patients [2], [18], [20]. ADEM is a monophasic illness with polymorphous clinical manifestations, that may pertain to motor, cerebellar, brainstem or myeloradicular involvement. In the absence of a specific biomarker, diagnosis may be made on MRI, which shows symmetrical multifocal white matter demyelinating lesions involving the brain and/or the spinal cord [4]. Being an immune-mediated disorder in nature, variable clinical outcomes in ADEM have been reported which were treated with either intravenous steroid or immunoglobulin therapy, however, no established treatment guidelines based on randomized clinical trials exist. Here we present two cases of patients with positive COVID-19 infection who developed neurological symptoms during the hospitalization.

## Case Report

### Case 1- Father

**Fig. 1:**
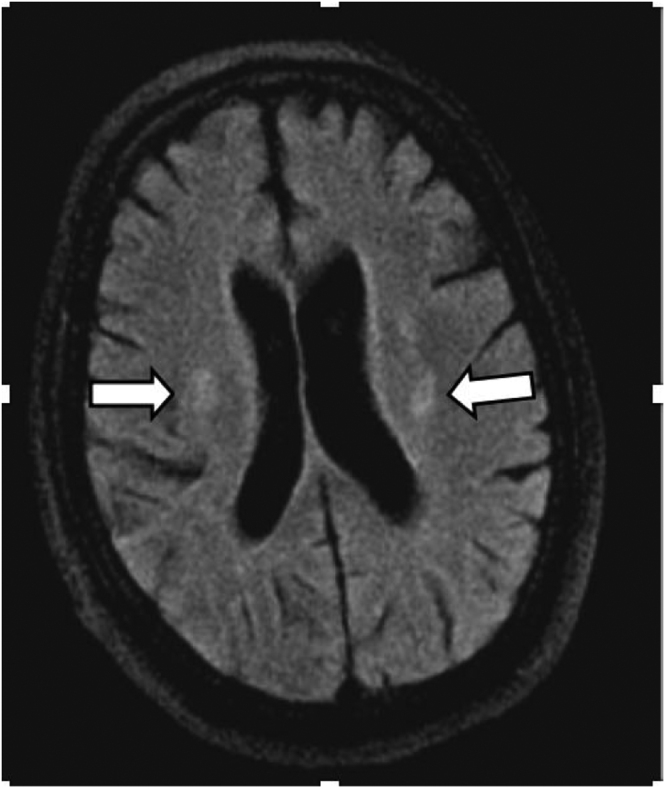
Initial non-enhanced MRI brain. Axial FLAIR sequence demonstrates multifocal symmetrical periventricular white matter hyperintensities (White arrows).

A 68-year-old Hispanic male with a longstanding history of multiple medical comorbidities, including diabetes mellitus, hypertension, coronary artery disease (CAD), and chronic obstructive pulmonary disease (COPD) presented to the hospital with shortness of breath, chest congestion, fever, headache, and sore throat for a week. He had no prior neurological illness. Chest x-ray revealed extensive bilateral patchy pulmonary airspace opacities. Nasopharyngeal swab for SARS-CoV-2 RNA was positive. Shortly after his admission, he was intubated due to respiratory failure, and was maintained on continuous sedation drips. He had a complicated course of hospitalization in which he developed COVID-19-associated acute respiratory distress syndrome (ARDS). Furthermore, he had septic shock which required vasopressors and antibiotics, he required hemodialysis due to renal failure, gastrointestinal bleeding occurred, and he developed a deep venous thrombosis (DVT) of the right lower extremity. On day 30, while off of sedation, he was found to have a grossly depressed mental status with neurological examination demonstrating an unresponsive patient with Glasgow Coma Scale of 3 (E1V1M1). Pupils were equal and reactive to light. Muscle tone was increased and there were 2 + deep tendon reflexes in biceps/triceps, and 1 + in the patellar tendon bilaterally. Extensor plantar response was present bilaterally as well. The remaining reflexes could not be elicited. MRI of the brain with and without gadolinium contrast revealed bilateral periventricular white matter hyperintense foci on fluid attenuated inversion recovery (FLAIR) sequence, without associated restricted diffusion, enhancement, or hemorrhage (Fig. 1). MRI cervical spine revealed no abnormalities in the spinal cord (Fig. 5). For stroke evaluation, an MR angiogram of the brain and neck along with a transthoracic echocardiogram were performed and all were normal. CSF analysis was done to rule out infectious process which demonstrated 3 white blood cells, 50 red blood cells, 28 mg/dl protein, and a glucose of 109 mg/dl. Serum glucose at the time of lumbar puncture is 142. IgG oligoclonal bands to assess for multiple sclerosis were not detected in the cerebrospinal fluid. CSF cultures and PCR panels (including COVID-19 RNA, HSV, VZV, EBV, and CMV) were negative. Anti- myelin basic protein antibody test was negative. EEG demonstrated diffuse slowing and a disorganized pattern. Ultrasound venous doppler of lower extremities showed a partial occlusive thrombus in the right femoral vein, but anticoagulation was not started due to his recent history of GI bleeding. CT chest demonstrated extensive bilateral interstitial thickening and fibrosis without pulmonary embolism. Serum testing for ANA, ANCA, HIV, syphilis, AFB were negative, and coagulation factors were within normal limits. He was started on 40 mg IV methylprednisolone on day 30 for the next few weeks. Follow-up MRI of the brain without contrast on hospital day 44 demonstrated stable size and distribution of the white matter signal changes in the brain (Fig. 3). His neurological and respiratory status improved, and by day 50 he was extubated, and rehabilitation therapy was initiated. Over the next few days, there was complete resolution of motor aphasia and his muscle strength improved to 4/5. By day 70, he was able to walk with assistance. He remains hospitalized at the time of writing this case report, and he requires broad spectrum antibiotic treatment for an infected pneumatocele that developed from complications of the COVID-19 infection.

**Fig. 2:**
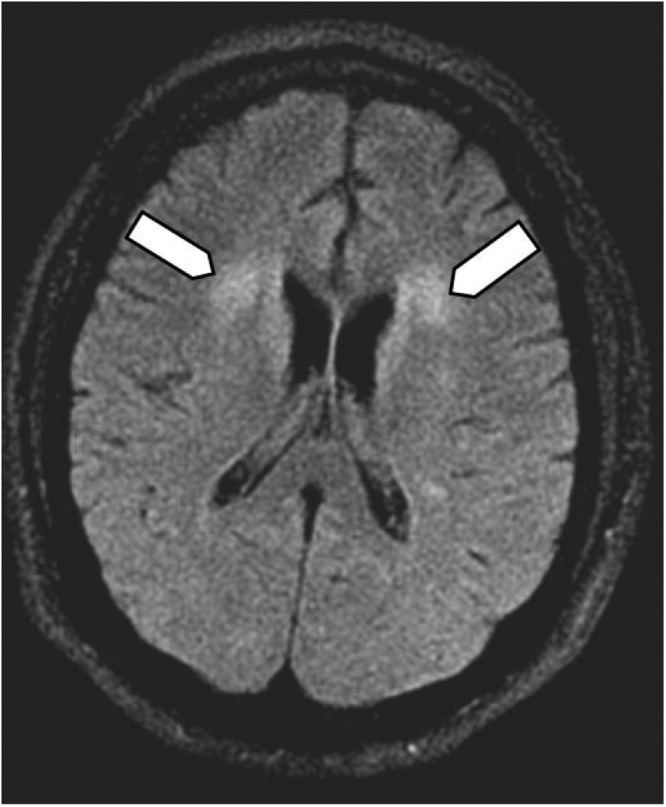
Initial non-enhanced MRI of the brain. Axial FLAIR sequence demonstrates bilaterally prominent periventricular white matter signal hyperintensities (white arrows).

**Fig. 3:**
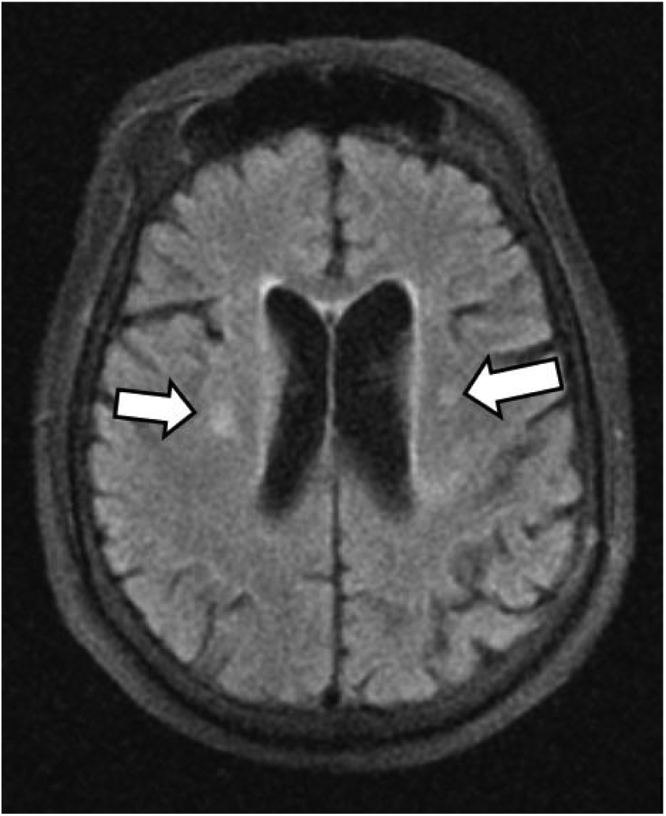
Follow-up non-enhanced MRI brain; Axial FLAIR sequence demonstrates stable size and distribution of white matter changes in the brain. (White arrows).

**Fig. 4:**
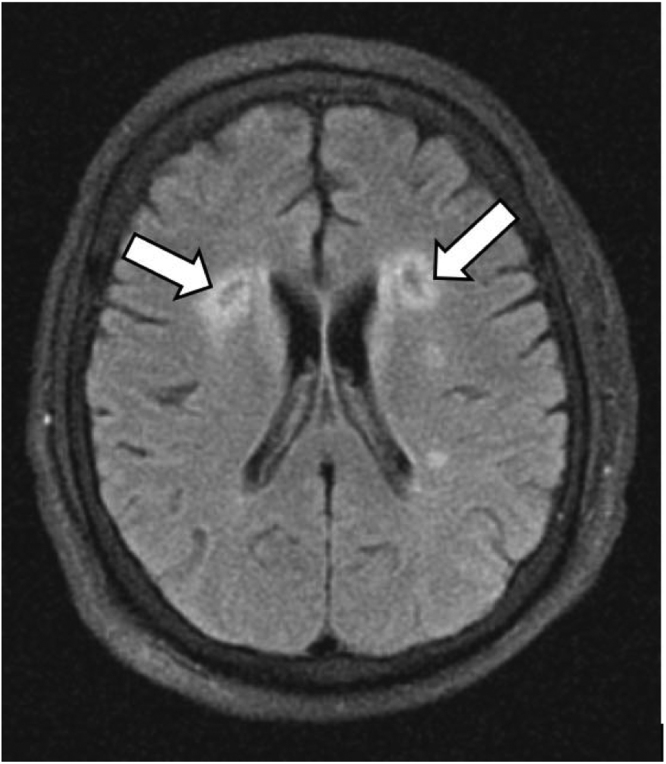
Follow-up non-enhanced MRI of the brain; Axial FLAIR sequence demonstrates central encephalomalacia in the lesions within the frontal lobe (White arrows).



### Case 2- Son

A 49-year-old Hispanic male, the son of the a forementioned patient, with past medical history of hypertension and hyperlipidemia presented to the hospital on the same day as his father with intermittent fever, dry cough, anosmia, and sore throat. He was febrile, tachypneic, hypotensive, and hypoxic requiring intubation and sedation. Nasopharyngeal swab for SARS-CoV-2 RNA was positive. He had a complicated course of hospitalization during which he developed ARDS from COVID-19 (which led to extensive pulmonary fibrosis and pneumatoceles), acute kidney injury (AKI) requiring hemodialysis (HD), multi-organ failure with septic shock, and gastrointestinal bleeding for which he was placed on a pantoprazole drip. He had been on several sedatives and all of them were discontinued on day 26 after sedation vacation protocol. On day 30, he was found to have a grossly depressed mental status with GCS 3 (E1V1M1). He was not on any paralytic agents other than a single dose of vecuronium which was used during his initial intubation. On physical examination, deep tendon reflexes showed a brisk response bilaterally in the upper and lower extremities. There was extensor plantar response bilaterally. There was a faint positive corneal and sensory response bilaterally, but he demonstrated a positive Doll's eye reflex. CT of the brain without contrast showed no acute intracranial process. MRI of the brain performed with and without IV gadolinium contrast showed bilateral multifocal periventricular white matter FLAIR signal hyperintensities (Fig. 2). There was no hemorrhage, restriction in diffusion, or contrast enhancement in any of these findings. MRI of the cervical spine with and without contrast on day 38 was normal (Fig. 6). CSF analysis was done to rule out an infectious process which revealed pink and hazy fluid with 9 white blood cells, 1100 red blood cells, 91 mg/dl proteins, and a glucose of 66 mg/dl. Serum glucose at the time of lumbar puncture is 138. CSF cultures and PCR panels including COVID-19 RNA, HSV, VZV, EBV, and CMV were negative. CSF was negative for oligoclonal bands. Anti-myelin basic protein antibody was done to rule out multiple sclerosis which came back negative. EEG demonstrated underlying theta activity throughout the tracing with no evidence of paroxysmal activity. Transthoracic echocardiogram showed mild mitral regurgitation without evidence of a thrombus. Serum testing of ANA, ANCA, HIV, syphilis, and coagulation factors were normal or negative. IV methylprednisolone 40 mg was initiated for the next few weeks. Follow-up MRI of the brain without contrast on day 49 showed stable size and distribution of the signal changes, but there were encephalomalacic changes within the lesions of the frontal lobes (Fig. 4). Clinically, his mental alertness continued to improve, and he was successfully extubated by day 60 and physical rehabilitation therapy was provided. There was complete resolution of his motor aphasia, and motor strength was partially improved with 3/5 strength of the bilateral lower extremities by day 70. IVIG therapy was not initiated at this time due to lack of evidence in our literature search for patients with COVID-19 pneumonia. Therefore, we continued with steroid therapy in anticipation of improvement of symptoms. He remains hospitalized at the time this case report was written, and he is still undergoing aggressive physical therapy and steroid management.

**Fig. 5:**
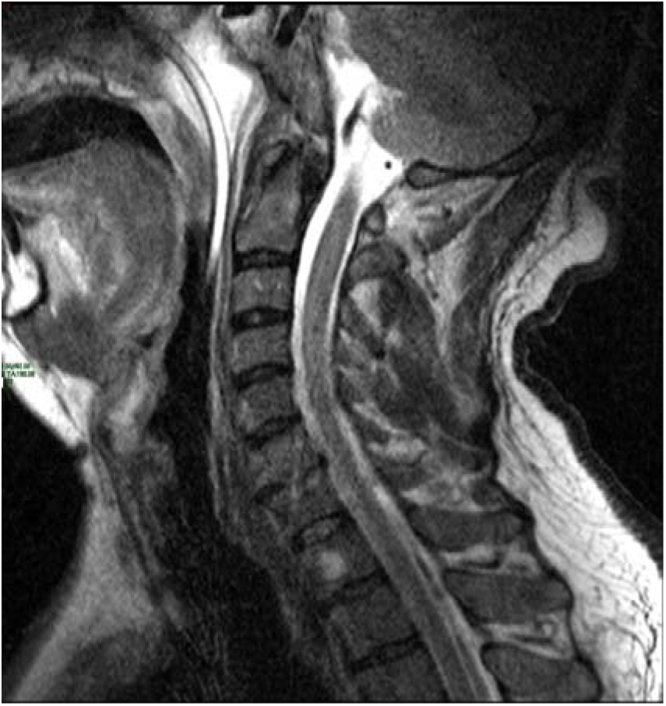
MRI cervical spine revealed no abnormalities in the spinal cord.

**Fig. 6:**
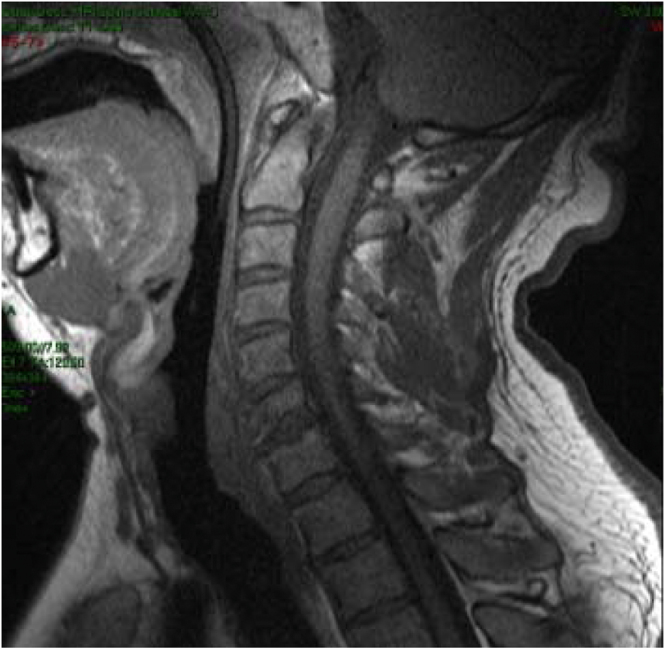
MRI cervical spine with and without contrast shows no evidence of cord compression and degenerative changes.

.

## Discussion

The World Health Organization (WHO) has declared SARS-CoV-2 infection a global pandemic on March 11th, 2020 [1]. Despite its most characteristic symptoms of respiratory distress, several neurological manifestations have been reported during the hospitalization in patients with severe COVID-19 infection [3], [8]. New data regarding the heterogeneity of neurologic manifestations of SARS-CoV-2 have been rapidly emerging [3]. The exact mechanism by which SARS-CoV-2 involves the brain and spinal cord is yet to be established; although, ACE-2 receptor-mediated injury to the neuronal and glial cells is widely considered as a putative mechanism of neurological injury causing demyelinating lesions in the brain and spinal cord [6], [8], [10]. Early detection of neurological deficits may improve treatment algorithms and potentially lead to better outcomes [5].

Strokes have been commonly reported in COVID-19 patients [19]. While our patients had risk factors for stroke, neurovascular imaging and echocardiograms excluded large vessel territory strokes in our patients. Various inflammatory/demyelinating disorders were considered as differential diagnoses and were excluded in our patients before arriving at our final diagnoses. Multiple sclerosis (MS) was within the main differential diagnosis because of predominant white matter affliction and due to familial involvement [21]. However, the absence of oligoclonal bands in the CSF, negative anti-myelin basic protein antibody, and lack of any prior episodes of neurological deficits made MS less likely a cause. Lack of spinal cord lesions and of optic neuritis excluded neuromyelitis optica spectrum disorders and transverse myelitis. Absence of brainstem involvement and no prior history of autoimmune disorders, such as Bechet’s disease or SLE, excluded autoimmune encephalitis which typically involves the brainstem. Acute hemorrhagic necrotizing encephalopathy has been recently reported in a middle-aged female with COVID-19 after a 3-day history of illness [22]. ADEM often develops following an upper respiratory usually of a viral cause including influenza, measles, CMV, herpes simplex virus, MMR and EBV, we ruled out the infectious etiology by CSF cultures which back negative, influenza testing is negative and blood, urine cultures were negative. Using clinical criteria, neuroimaging, and excluding other possible causes, ADEM was diagnosed. ADEM is an inflammatory demyelinating disorder which can develop at any age, but children are more commonly affected than adults [16], [17]. Anti-myelin oligodendrocyte glycoprotein (MOG) autoantibody is known to be positive in CNS autoimmune disorders which includes ADEM, which would have been supportive of the clinical diagnosis. But this testing was unavailable to us. There are very few cases of ADEM that have been reported in the literature associated with COVID-19 viral infection [2], [14]. It is not an inherited condition and there are no reported cases of ADEM occurring in more than one family member to our knowledge. Here we demonstrate a unique presentation of familial cases of COVID-19 associated with ADEM in a hospitalized father and son.

Most neurological manifestations of ADEM typically occur around 2–4 weeks following viral illness or vaccination [11], [12]. In our patients, ADEM manifested late, around day 30 of hospitalization. The exact time of onset of neurological symptoms was difficult to ascertain as the patients were intubated and on paralytic agents. Radiologically, T2-weighted FLAIR sequences on MRI best depict the manifestations of ADEM [4], [7], [9]. In this case report, the initial MRI axial FLAIR sequence demonstrated prominent, bilateral periventricular white matter signal hyperintensities (Fig. 1, Fig. 2) based on which ADEM was suspected. On follow-up MRI in these patients, it was evident that the lesions in the father were stable, while some lesions in the son developed encephalomalacia (Fig. 3, Fig. 4 respectively), suggesting severe neuroglial tissue loss due to inflammation in the latter. Notably, the severity of the COVID-19 infection course in the son was worse compared to his father.

Methylprednisolone is the initial treatment of choice for ADEM, although no controlled trials have established its superiority over other treatments. The outcome of ADEM using IV methylprednisolone is generally good, especially in children, with 57–81% of patients having complete recovery [16], [17]. Unfortunately, high dose steroids have been considered controversial in the treatment of COVID-19 at this time, with some studies even suggesting that high doses of steroids may be detrimental in COVID-19 disease course [13], [15]. We did start both of our patients on Dexamethasone 6 mg for total 10 days as per the RECOVERY trail (ClinicalTrials.gov number, NCT04381936) [23], we mentioned the treatment summary in the timeline of detailed events during hospitalization. A recent case-study examined COVID-19-associated ADEM in a 51-year-old who had classic features and MRI findings. When treated with IV methylprednisolone, she was found to have a favorable response [18]. Therefore, it is currently unclear if high-dose steroids should be used in the treatment of patients presenting with this rare neurologic manifestation of SARS-CoV-2. Both of our patients were initially started on medium-dose IV methylprednisolone with tapering doses resulting in significant clinical improvement in the first case of the father. However, the second case with the son did not demonstrate similar clinical improvement and he developed encephalomalacia. Despite these improvements with IV steroids, more research is needed to determine proper management strategies for ADEM in the setting of COVID-19. IVIG is another treatment for ADEM patients, however, to our understanding it was not well-studied in COVID-19 infections.

COVID-19 associated ADEM in this father and son is unique, raising the possibility of an underlying genetic component, and to the best of our knowledge, there has been no previous documentation of familial involvement of ADEM. Both of our patients did not live together. However, 10–12 days before the hospitalization they both participated in a family gathering which contributes to disease development. While it is possible that shared-environmental factors or other non-genetic factors also contribute to disease development, this case series of a father and son with ADEM following COVID-19 may be an indicator of an underlying genetic susceptibility. At the 6-month follow up, there were no recurrent symptoms in these patients and able to walk with no assistance so, we do not consider doing WGS for our patients. As this pandemic unfolds, more information and research will be made available to identify risk factors for neurological complications in COVID-19.

## Conclusion

This case series of father and son with severe COVID-19 infection-related complications raises the possibility of a shared genetic susceptibility towards developing ADEM. The neurological outcome in these two hospitalized patients appeared to be dependent on the severity of the COVID-19 infection. As the COVID-19 pandemic unfolds, more information regarding the underlying risk factors, treatment, and prognosis will help us in treating these afflicted patients and their neurological complications.

## Funding Statement

“The author(s) received no specific funding for this work.”

## Patient consent

No written consent has been obtained from the patients as there is no patient identifiable data included in this case report.

## Data Availability

Data AvailabilityData sharing not applicable to this article as no datasets were generated or analyzed during the current study.
